# Do risk profiles moderate the relation between age of onset of disruptive behaviour and two types of externalising problems among adolescents admitted to secure residential care?

**DOI:** 10.1186/s13034-021-00364-8

**Published:** 2021-02-26

**Authors:** Miranda G. W. G. Knoops, Ilja L. Bongers, Elisabeth A. W. Janssen-de Ruijter, Chijs van Nieuwenhuizen

**Affiliations:** 1 GGzE Centre for Child and Adolescent Psychiatry, DP 8001, PO BOX 909, 5600 AX Eindhoven, The Netherlands; 2 Tilburg University, Tranzo, Scientific Center for Care and Wellbeing (Tranzo), Tilburg, The Netherlands

**Keywords:** Risk profiles, Age of onset, Adolescents, Secure residential care, Forensic psychiatry

## Abstract

**Background:**

Adolescents with externalising problems in secure residential care differ in age of onset of disruptive behaviour and in cumulative risks in several domains. In order to reduce negative consequences of externalising behaviour for society and the adolescents themselves, it is important to gain more insight into the complexity and heterogeneity of disruptive behaviour in these adolescents. To look beyond the influence of single risk factors, the aim of this study is to investigate the moderator effect of co-occurring risk factors in multiple domains on the relation between age of onset of disruptive behaviour and two types of externalising problems in adolescence.

**Methods:**

Retrospectively collected data of 225 adolescents admitted to secure residential care were analysed. The four risk profiles were based on co-occurring pre-admission risk factors in four domains, which were identified in a previous study by latent class analysis. Multiple regression models were used to test whether the independent variable age of onset and dummy-coded moderator variable risk profiles had statistically significant associations with aggressive behaviour and rule-breaking behaviour of the adolescents, as reported by professional caregivers in the first months of admission.

**Results:**

Risk profiles moderated the relation between age of onset of disruptive behaviour and rule-breaking behaviour. Adolescents with childhood-onset disruptive behaviour within the risk profile with mainly family risks showed more rule-breaking behaviour in the first months of their admission to secure residential care than adolescents with an onset in adolescence within the same risk profile. Risk profiles, however, did not moderate the relation between age of onset of disruptive behaviour and aggressive behaviour.

**Conclusion:**

Heterogeneity of aggressive and rule-breaking behaviour was established in this study by finding differences on these two types of externalising behaviour between the childhood- and adolescence-onset groups and between the four risk profiles. Furthermore, risk profiles moderated the effect between age of onset of disruptive behaviour and rule-breaking behaviour—not aggressive behaviour—in adolescents admitted to secure residential care. While respecting the limitations, adolescents’ childhood-onset disruptive behaviour within a profile with mainly family risk factors appear to be distinctive from adolescents with adolescence-onset disruptive behaviour within the same risk profile.

## Background

Adolescents showing disruptive behaviour or externalising problems in childhood, compared with adolescents who are not familiar with disruptive behaviour before adolescence, have a two- to threefold increased risk for developing a criminal career in adulthood [1]. Disruptive behaviour in childhood is not only related to delinquent behaviour in adolescence; children with disruptive behaviour also often develop into adults who are poorly adjusted [2]. Furthermore, disruptive behaviour in childhood is related to low social competence, peer difficulties, school failure, early school dropout, substance abuse, unemployment, and incarceration in later life [1–4].

The developmental taxonomy of antisocial behaviour [5, 6] states that the onset of disruptive behaviour distinguishes between adolescents with aggressive and adolescents with rule-breaking behaviour. Children with early onset of disruptive behaviour and life course persistent disruptive behaviour exhibit more antisocial and aggressive behaviour during adolescence than those who develop disruptive behaviour during adolescence. The disruptive behaviour of the latter group is mainly characterised by rule-breaking behaviour and authority conflicts [5–7]. The developmental taxonomy of Moffitt [5, 6] primarily pertains to disruptive behaviour problems, whereas a majority of adolescents with disruptive behaviour are also diagnosed with a psychiatric disorder [8]. Hence, the relation between age of onset of disruptive behaviour and type of externalising problems during adolescence also applies to adolescents with comorbid complex psychiatric problems.

Adolescents with various types of externalising problems, such as aggressive behaviour and rule-breaking behaviour, not only differ in the age of onset of the disruptive behaviour and perseverance of the externalising problems, their risk factors are also diverse [6, 9–11]. Prior research shows that adolescents with early onset of disruptive behaviour have more risk factors in the family domain, such as inadequate parenting and family conflict. These factors are associated with predisposing familial, neuropsychological deficits, and hyperactive temperament, which interact with environmental factors that lead to more severe externalising problems in adolescence and adulthood [10, 12, 13]. Otherwise, adolescents with late onset disruptive behaviour have more risk factors in the peer domain, such as peer rejection and delinquent peers [9, 12, 14–17]. Peer rejection and deviant peer affiliation are a sequential process, where peer rejection is more prominent in childhood and deviant peer affiliation is more on the foreground in adolescence. Studies suggest a strong socializing influence of peers during adolescence, this influence of deviant peers applies particularly to adolescents with late starting and adolescence-limited disruptive behaviour [5, 18–20]*.* Left untreated, these early and late onset externalising problems often become persistent, resulting in development of antisocial behaviour and delinquency in adolescence and adulthood.

As regard to risk factors, the cumulative risk hypothesis [21, 22] postulates that the accumulation of risk factors, instead of the presence or absence of particular risk factors, is associated with an increased prevalence of disruptive behaviour. Furthermore, exposure to an accumulation of risks in multiple domains, rather than only in the individual, family, peer or school domains, increases the likelihood of developing disruptive behaviour [23, 24]. Combining the established predictors of later problems—that is, the accumulation of risks in multiple domains and the early onset of problems—could gain more insight into the complexity and heterogeneity of disruptive behaviour. In the end, this insight might reduce the negative consequences for society and for the adolescents themselves.

Among adolescents admitted to residential care, disruptive behaviour is highly prevalent; clinical externalising problems occur in 60–79% of the adolescents at admission, and many adolescents (39–49%) has been classified with a disruptive behaviour disorder prior to admission [25–28]. These vulnerable adolescents also often have had to deal with various adverse circumstances from an early age. In a previous study, heterogeneity in this population was confirmed by finding four subgroups of adolescents with distinct patterns of co-occurring risk factors in multiple domains [29]. Two subgroups were found with adolescents with many risk factors in multiple domains and two groups with adolescents with fewer (but still several) risk factors in single domains were found. Since adolescents in this population have not individual but instead multiple risk factors in several domains, it is preferable to look beyond the influence of single risk factors as was done in previous research. Using a person-centered approach, the insight into the influence of co-occurring risk factors on the relationship between age of onset and problem behaviour in this population of adolescents admitted to secure residential care can be investigated. This may be the next step to better understand the complexity and heterogeneity of disruptive behaviour in this vulnerable population with high occurrence of disruptive behaviour, whereby more information can be obtained about the varying causes of this behaviour. In addition, this insight could improve the prevention and treatment of externalising problems. Therefore, the aim of this study is to investigate the moderator effect of the four risk profiles from a previous study [29] on the relation between age of onset of disruptive behaviour and two types of externalising problems in adolescence.

## Methods

### Setting

This study was conducted at the Catamaran, a hospital for youth forensic psychiatry and orthopsychiatry in the Netherlands. This secure residential setting offers intensive multidisciplinary treatment to males and females (13 up to and including 23 years old) who are sentenced under Dutch juvenile civil law or criminal law, or are admitted voluntarily. Measures under the Dutch juvenile civil law are applied to adolescents who pose a risk to themselves or to others. Dutch juvenile criminal law comprises the treatment and rehabilitation of adolescents^1^ who have committed offences and suffer from major psychiatric problems. Irrespective of the type of measure, all the patients in this hospital suffer from severe multiple problems in multiple life areas.

A multidisciplinary team of psychologists, psychiatrists, family therapists, social workers, and staff workers provides the intensive residential treatment at the Catamaran. This treatment comprises for instance aggression regulation therapy, psychomotor therapy, systemic therapy, psychotropic medication, cognitive behavioural therapy, job training, and education. Since 2014, the Catamaran has been awarded a certificate for highly specialised care for patients with serious complicated mental health problems.

### Participants

The sample comprised 274 male patients who were admitted to the Catamaran between January 2009 and October 2017. Because 96% of the admitted adolescents were male, only male patients were included. In addition, 49 patients were excluded for the sample for four reasons: (a) 25 patients objected to the use of their data for research purposes; (b) seven patients were excluded because of missing data on age of onset of disruptive behaviour; (c) eight patients were excluded because they had no problem behaviour before admission; and (d) nine patients were excluded because of missing data on their type of externalising problems. Hence, this resulted in a final sample of 225 patients. To assess significant differences between the included (*n* = 225; 90%) and excluded sample (*n* = 24, 10%) the classification to the risk profiles was compared. There was found no significant difference between the included and excluded patients.

In the final sample (*N* = 225), the mean age at admission was 16.8 years (range 13–23, SD = 1.9). Almost half the sample (48.9%) were detained under Dutch juvenile criminal law, 33.3% under Dutch juvenile civil law, and 17.8% were admitted voluntarily. In 68.7% of the participants, both parents were born in The Netherlands. A minority of the patients (28.4%) had not been convicted before admission. Among the patients with previous convictions, moderate violent offences (e.g., threatening with violence, illegal weapon ownership) and property offences without violence (e.g., theft without violence, extortion) were most common, 48.9 and 42.2%, respectively. As for the psychiatric background, most of the DSM-IV-TR disorders [30]—stated at the start of admission by the clinical coordinator in the (electronic) patient file—were in the category ‘disorders usually first diagnosed in infancy, childhood, or adolescence’, in particular, disruptive behaviour disorders (45.3%), autism spectrum disorders (37.8%) and attention deficit/hyperactivity disorders (24.9%). Other common Axis-I disorders were substance disorders (25.8%) and reactive attachment disorders (15.1%). On Axis-II, 8.9% of the adolescents had the classification mental retardation and 4.9% a personality disorder. Detailed background characteristics are displayed in Table 1.

**Table 1:** Differences between the risk profiles in age of onset, externalising type of behaviour and demographic characteristics (N = 225)

	Overall mean or percentage	Three-domain risk profile (*n* = 95)	Four-domain risk profile (*n* = 57)	Profile with mainly peer risks (*n* = 41)	Profile with mainly family risks (*n* = 32)	F/χ^2^	*p*
Age at admission in years	16.8	17.1	17.3	16.1	15.8	7.29	0.000
Early onset	56%	46%	60%	56%	81%	12.56	0.006
Aggressive behaviour	13.0	12.6	12.5	11.8	16.2	2.42	0.067
Rule-breaking behaviour	10.4	11.8	10.4	7.9	9.7	5.51	0.001
Previous delinquent behaviour^a^							
No conviction	28.4%	20.0%	15.8%	46.3%	53.1%	22.83	0.000
Drug offence	6.2%	11.6%	1.8%	2.4%	3.1%	6.67	0.061
Vandalism (property)	27.1%	35.8%	36.8%	9.8%	6.3%	21.27	0.000
Property offence without violence	42.2%	51.6%	64.9%	7.3%	18.8%	46.93	0.000
Moderate violent offence	48.9%	54.7%	66.7%	29.3%	25.0%	22.29	0.000
Violent property offence	24.0%	34.7%	29.8%	7.3%	3.1%	23.25	0.000
Serious violent offence	7.1%	11.6%	8.8%	0%	0%	8.69	0.023
Sex offence	8.9%	4.2%	8.8%	22.0%	6.3%	9.70	0.015
Manslaughter	3.6%	1.1%	7.0%	4.9%	3.1%	4.21	0.182
Arson	0%	0%	0%	0%	0%	–	–
Murder	1.8%	1.1%	3.5%	2.4%	0%	1.90	0.609
Axis-I classification of DSM-IV-TR^b,c^							
Disruptive behaviour disorder	45.3%	53.7%	56.1%	24.4%	28.1%	16.59	0.001
Autism spectrum disorder	37.8%	38.9%	21.1%	70.7%	21.9%	28.73	0.000
Substance disorder	25.8%	37.9%	35.1%	0%	6.3%	37.31	0.000
Attention deficit/hyperactivity disorder	24.9%	32.6%	15.8%	22.0%	21.9%	5.72	0.122
Reactive attachment disorder	15.1%	4.2%	26.3%	9.8%	34.4%	24.37	0.000
Anxiety disorder	8.4%	3.2%	12.3%	4.9%	21.9%	11.44	0.006
Schizophrenia or other psychotic disorder	6.2%	8.4%	10.5%	0%	0%	7.23	0.048
Mood disorder	6.2%	6.3%	5.3%	9.8%	3.1%	1.38	0.723
Other disorder usually first diagnosed in infancy, childhood or adolescence	5.3%	5.3%	0%	7.3%	12.5%	7.22	0.041
Axis-II classification of DSM-IV-TR^b^							
Personality disorder	4.9%	2.1%	12.3%	0%	6.3%	8.73	0.016
Mental retardation	8.9%	10.5%	14.0%	4.9%	0%	6.14	0.098

^a^Classification of Van Kordelaar [58]

^b^Only DSM-IV-TR classifications with a prevalence of > 5% are displayed

^c^Due to comorbidity, percentages of DSM-IV-TR classifications do not sum up to 100

### Measurements

#### Types of externalising problems at the start of admission

The type of externalising problems was operationalised by means of the syndrome scales ‘rule-breaking behaviour’ and ‘aggressive behaviour’ of the Child Behaviour Checklist (CBCL; [31]). The CBCL is a questionnaire containing 113 items that assesses behavioural and emotional problems in the previous six months. Professional caregivers working in close contact with the adolescents filled out the CBCL within half a year after admission. All items are scored on a three-point Likert scale (0 = not true, 1 = somewhat of sometimes true, and 2 = very of often true). The CBCL is a widely used, standardised, norm-based questionnaire with extensive evidence for reliability and validity [31–33]. In the present study, the internal consistency of the syndrome scale ‘rule-breaking behaviour’ was 0.79 and that of the syndrome scale ‘aggressive behaviour’ was 0.90.

#### Age of onset

Age of onset of disruptive behaviour was operationalised by means of the item ‘onset of problem behaviour’ of the Juvenile Forensic Profile (JFP; [34, 35]). The JFP assesses risk factors and is based on existing internationally- and nationally-validated instruments for measuring problem behaviour and risk assessment [36, 37]. The psychometric properties of the JFP are satisfactory [35].

The item ‘onset of problem behaviour’ refers to the first start of problem behaviour, for example, being sent from school or contact with police or social workers due to problem behaviour of the child. The item is measured on a three-point scale: 0 = no mention of earlier problem behaviour, 1 = problematic behaviour first mentioned after moving from primary school to secondary school, when the adolescent is 12 years or older, 2 = clearly visible signs of severe problem behaviour before the 12th year of life. Patients with score 0 were excluded for the sample. For all other patients, the age of onset of the sample was recoded into 2 = childhood onset, and 1 = adolescence onset.

#### Risk profiles

In this study, we used the risk profiles previously found in a study on the same population [29]. Eleven risk factors in individual, family, peer, and school domains were derived from the Structured Assessment of Violence Risk in Youth (SAVRY; [38]) and the JFP. The individual domain comprised hyperactivity, cognitive impairment and history of drug abuse. The family domain consisted of exposure to violence in the home, childhood history of maltreatment and criminal behaviour of family members. The peer domain contained three risk factors: peer rejection, involvement in criminal environment and lack of secondary network. The two risk factors in the school domain were low academic achievement and truancy.

Through latent class analysis (LCA), by means of Latent GOLD 5.0 [39, 40], four risk profiles were identified. All adolescents in the present study’s sample are assigned to these four risk profiles. The first profile comprised adolescents with many risk factors in the individual, peer, and school domains (three-domain risk profile), for example drug abuse, involvement in criminal environment, and truancy. The second profile comprised adolescents with many risk factors in the individual, family, peer, and school domains (four-domain risk profile), such as drug abuse, childhood history of maltreatment, and lack of a secondary network. The third profile concerned adolescents with risk factors mainly in the peer domain (profile with mainly peer risk factors), particularly peer rejection. The fourth profile represented adolescents with risk factors mainly in the family domain (profile with mainly family risk factors), for example exposure to violence in the home and childhood history of maltreatment.

### Procedure

Data were collected using (electronic) patient files and routine outcome questionnaires of the adolescents admitted to the hospital. Because the hospital treats adolescents up to 23 years old, the CBCL was also completed for adolescents older than 18 years old [41]. Mental health professionals used routine outcome monitoring (ROM) as input for treatment and evaluation. From the ROM database, data from the first completed CBCL were selected. Within half a year after admission of the adolescent to the hospital, the professional caregiver who was in closest contact with the adolescent was asked to complete the CBCL.

Scoring of the historical items of the JFP took place one month after admission (when all required documents were collected). All possible sources pertaining to the patient’s life before admission (e.g. diagnostic reports from psychologist and psychiatrists, criminal records, treatment plans from previous settings, and judicial documents) were used. Scoring of the JFP took place by officially-trained researchers and trainees under supervision. The JFP was completed by means of consensus scoring until an interrater reliability of at least 80% was achieved [29].

Risk profile membership were partly derived from the data collection of a previous study [29]. The datasets of this aforementioned study and the present study overlap with regard to 155 patients. Seventy patients were admitted to the hospital after the data collection of the previous study. They were posterior assigned to their appropriate risk profiles.

The Dutch Law on Medical Treatment Agreement, article 7: 458 states that scientific research is permitted without the consent of the patient if active informed consent is not reasonably possible, or if the type and aim of the study does not require such permission. Additionally, the law states that scientific research without the active consent of the patient is only permitted under three conditions: (1) the study is of general interest; (2) the study cannot be conducted without the requested information; and (3) the participant has not expressly objected to the provision of the data. The current study satisfied these conditions. Patient anonymity was guaranteed by assigning a research number to each patient. Twenty-five patients explicitly objected to the provision of their data for research purposes and were therefore excluded. To ensure a comprehensive assessment, it was been discussed thoroughly by the Ethics Review Board of Tilburg University, and by the science committee of GGzE, the Institute of Mental Health Care. Their decision was that no further ethical assessment was needed. Hence, the present study was conducted in accordance with the prevailing medical ethics of the Netherlands. In addition, the procedures were in accordance with the 1964 Declaration of Helsinki and its later amendments or comparable ethical standards.

### Statistical analysis

In Latent GOLD, the estimated LC model parameters of risk membership as calculated in the previous study [29] were used to classify new observations. For this purpose, classification probabilities were reformulated as a set of logistic equations, which in Latent GOLD are referred to as scoring equations. These equations were applied to the new observations.

Data analyses were performed using SPSS version 19. For all analyses, a significance level of p < 0.05 was applied. Descriptive statistics were used to describe the characteristics of the sample. To study possible differences between the risk profiles, chi-square analyses and analyses of variance (ANOVA) were used, whereby Bonferroni correction was applied to correct for multiple testing.

The moderation analyses were conducted using the PROCESS macro [42, 43] for SPSS, version 23. Multiple regression models tested whether the independent variable age of onset and dummy-coded moderator variable risk profiles had statistically significant associations with the aggressive behaviour and rule-breaking behaviour reported by the professional caregiver. For the independent variable age of onset, the adolescence-onset group was the reference category and for the moderator variable risk profiles, the four-domain risk profile was the reference category. The statistical interaction between the independent variables (age of onset and risk profiles) indicated whether the relation between age of onset of disruptive behaviour and type of externalising problems were moderated by risk profiles. In order to probe the interactions, analyses using the Johnson-Neyman technique were conducted for all four outcomes variables [43]. The Johnson-Neyman technique calculates the statistical significance of the effect of an independent variable (in the current study, age of onset), for all values of the moderator variable (in the current study, the risk profiles), and plotted in a figure for each outcome measure.

## Results

All adolescents were classified to the four risk profiles: 95 adolescents were classified to the three-domain risk profile (42%), 57 adolescents to the four-domain risk profile (25%), 41 adolescents were classified to the profile with mainly family risks (18%), and 32 adolescents to the profile with mainly peer risks (14%). Differences between the four profiles were found in age at admission, age of onset, rule-breaking behaviour, and previous criminal behaviour (see Table 1). Adolescents with the four- and three-domain risk profiles had more often committed offences before admission. Moreover, they were more often classified with disruptive behaviour disorders, substance disorders, and/or schizophrenia or other psychotic disorders compared with adolescents with the other two risk profiles. Main differences between the adolescents with these four- and three-domain risk profiles were the percentage of autism spectrum disorders (which was higher in the three-domain risk profile), the percentage of reactive attachment disorders (which was higher in the four-domain risk profile), and the presence of family risks in the four-domain risk profile. The similarities between adolescents with the two other profiles with fewer risk factors (profile with mainly peer risks and profile with mainly family risks) were the higher percentages of no previous convictions and the younger age at admission to the Catamaran. Differences between adolescents with these two risk profiles were mainly found in their psychopathology: adolescents with the profile with mainly peer risks were most often classified with autism spectrum disorders, and adolescents with the profile with mainly family risks were more often classified with reactive attachment disorders and anxiety disorders. Furthermore, a majority of the adolescents (81%) with the profile with mainly family risks had an early onset of problem behaviour as opposed to 46% with the three-domain risk profile. In addition, adolescents with the profile with mainly peer risks had less rule-breaking behaviour in the first months of admission compared with adolescents with the three-domain risk profile.

Rule-breaking behaviour at admission did not significantly differ between adolescents with childhood-onset and adolescence-onset of disruptive behaviour, whereas aggressive behaviour at admission significantly differed between patients with childhood-onset and adolescence-onset of disruptive behaviour (see Table 2). To test the moderator effect of the risk profiles on the relation between age of onset of disruptive behaviour and the type of externalising behaviour during adolescence, multiple regression analyses were conducted for aggressive behaviour and rule-breaking behaviour with adolescents with a four-domain risk profile and childhood onset as reference categories (see Table 3). The overall fit of the model was significant. However, only the model for rule-breaking behaviour had a significant moderating effect of risk profiles. The overall model for rule-breaking behaviour explained 11% of the variance (model R^2^ = 0.11, F(7, 217) = 3.73*; p* < 0.001). The moderation effect is significant and explains 3% of the variance (test unconditional interaction effect R^2^ = 0.03, F(3, 217) = 2.73*; p* < 0.05).

**Table 2:** Differences between childhood onset and adolescence onset in externalising type of behaviour and demographic characteristics (N = 225)

	Childhood onset	Adolescence onset	F	*p*
Aggressive behaviour	14.9	10.4	20.696	0.000
Rule-breaking behaviour	10.6	10.2	0.215	0.643

**Table 3:** Results multiple regression models for the externalising type of behaviour during adolescence

	Aggressive behaviour			Rule-breaking behaviour		
Model						
F-test (p-value)	4.61 (.00)			3.73 (.00)		
R2	0.13			0.11		
Test unconditional interaction						
F-test (p-value)	2.26 (0.08)			2.73 (0.04)		
R2	0.03			0.03		

	Β	SE	P	B	SE	P
Constant	10.70	1.50	0.00	10.96	1.08	0.00
Age of onset^a^						
Childhood onset	3.10	1.95	0.11	− 0.90	1.40	0.52
Risk Profile^b^						
Three-domain risk profile	0.15	1.81	0.94	0.51	1.30	0.69
Profile with mainly family risks	− 4.36	3.30	0.19	− 6.79	2.38	0.00
Profile with mainly peer risks	− 0.36	2.27	0.87	− 3.07	1.63	0.06
Interaction						
Childhood onset X three-domain risk profile	0.74	2.45	0.76	1.59	1.76	0.37
Childhood onset X profile with mainly family risks	9.03	3.80	0.02	7.69	2.74	0.01
Childhood onset X profile with mainly peer risks	− 0.52	2.89	0.86	0.92	2.15	0.67

^a^Reference category = the adolescence-onset group

^b^Reference category = the four domain-risk profile

**Fig. 1:**
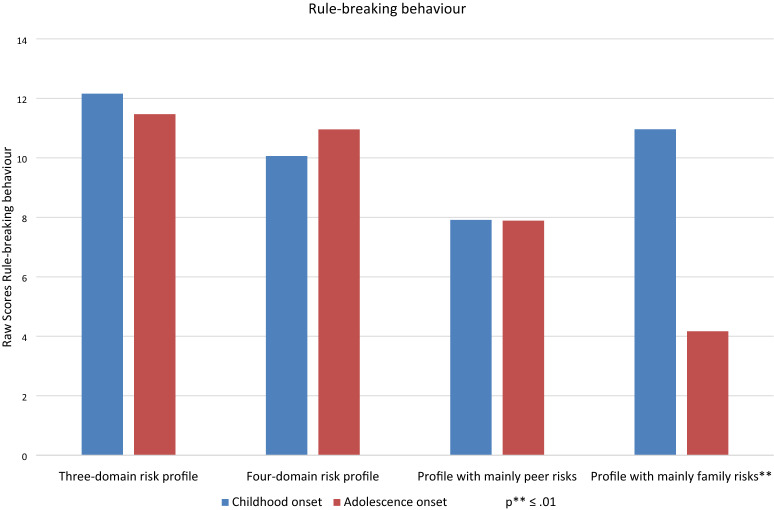
The moderating effect of risk profiles

As for the moderator effect of the risk profiles on the relation between age of onset and rule-breaking behaviour, rule-breaking behaviour does not differ significantly between the childhood-onset and adolescence-onset group. Only the family risk profile differed significantly from the reference category four-domain risk profile, there is a significant moderating effect on the relation between age of onset and rule-breaking behaviour (see Fig. 1). For this risk profile, the childhood-onset group showed significantly more rule-breaking behaviour (mean = 11.0) than the adolescence-onset group (mean = 4.2).

## Discussion

The high prevalence of disruptive behaviour among adolescents admitted to secure residential care was acknowledged in the present study. Furthermore, the heterogeneity of aggressive and rule-breaking behaviour was also established. Rule-breaking behaviour was equally prevalent in the childhood-onset group and the adolescence-onset group, but differed between the risk profiles. Adolescents with mainly peer risks had less rule-breaking behaviour in the first months of admission compared with adolescents with the three-domain risk profile. Aggressive behaviour was of similar occurrence in the four risk profiles, though it was more common in the childhood-onset group than the adolescence-onset group. Moreover, risk profiles moderated the relation between age of onset of disruptive behaviour and rule-breaking behaviour in adolescents admitted to secure residential care. According to professional caregivers, adolescents with risk factors mainly in the family domain and childhood-onset disruptive behaviour showed more rule-breaking behaviour in the first months of admission than adolescents with the same risk profile and adolescence-onset disruptive behaviour. Regarding aggressive behaviour, no moderating effect of risk profiles was found.

The only moderating effect on the relationship between age of onset and rule-breaking behaviour was found for the profile with mainly family risks. This group—which is 18% of the present study population—comprised adolescents who more likely had an early onset of problem behaviour, less likely had previous convictions compared with the three- and four-domain risk profiles, and who were often classified with a reactive attachment disorder or an anxiety disorder compared with adolescents with other profiles. The childhood-onset group within the risk profile with mainly family risk factors showed more rule-breaking behaviour at their start of admission to secure residential care than the adolescence-onset group within this profile. This result complements previous findings that adolescents with early onset of disruptive behaviour have more risk factors in the family domain (e.g., 6, 11). In the present study, a moderating effect was only found for the risk profile with mainly family risks (and without risk factors in other domains), and not for the four-domain risk profile in which risk factors were present in the family, peer, school, and individual domains (which was used as a reference category). This discrepancy highlights the importance of the use of a person-centered approach that goes beyond the influence of single risk factors.

The level of rule-breaking behaviour among adolescents with the three-domain risk profile, the four-domain risk profile, and the childhood-onset group within the family risk profile is found comparable high. Thus, the only significant difference is that adolescents with an adolescence onset and a risk profile with mainly family risks showed less rule-breaking behaviour at the start of admission to secure residential care compared with adolescents with a childhood-onset within the same risk profile. This difference can be explained by the psychopathology of the adolescents within this profile. These adolescents are relatively often diagnosed with a reactive attachment disorder and impaired attachment is related to authority conflict in adolescence [44]. Patterson et al. [45, 46] suggested that children with early onset and life course persistent disruptive behaviour are stuck with both temperamental risk factors and coercive parenting in childhood. In contrast, children with developing adolescence-limited disruptive behaviour in adolescence will be less socially skilled, but have experienced no major temperamental risk factors and only marginally family risks in childhood [47]. The relationship between temperamental risk factors and family risks in childhood, on the one hand, and childhood-onset disruptive behaviour, on the other hand, are in line with the findings of this study.

A striking finding of this study is that there was no moderating effect found for adolescents with the four-domain and three-domain risk profiles. Despite the cumulative risk hypothesis, the many risk factors in multiple domains in these adolescents had no added value in explaining externalising problems above the age of onset [21, 22]. Adolescents who experienced multiple risk factors from childhood onwards were relatively often diagnosed with various comorbid disorders [29]. Adolescents with multiple risk factors have been familiar with disruptive and aggressive behaviour for so long, that they hold beliefs that can normalise aggression [48]. In addition, they are more likely to use anger to cope, are more impulsive [49], and are less socially competent [50] than adolescents with single risk factors.

Another possible explanation for the absence of a moderator effect of the four-domain and three-domain risk profiles is the difference in the various related psychiatric disorders. Adolescents with these risk profiles are more often diagnosed with schizophrenia or psychotic disorder, substance disorder, or disruptive behaviour disorder than adolescents with the profile with mainly family risks. This serious psychiatric vulnerability can be the cause of both the presence of risk factors and the onset of problem behaviour and is not expected to have a moderating effect on the relation between age of onset of disruptive behaviour or rule-breaking problems during adolescence. The absence of the effect of the four-domain and three-domain profiles could possibly be an expression of a serious psychiatric vulnerability with a broad range of symptoms transcending diagnostic domains which could be considered as the General Factor of Psychopathology (p-factor), a variable associated with more life impairment and worse developmental histories [51]. This ‘p-factor’ seems to be stable during the development through childhood and adolescence, however, further research is needed to establish the developmental trajectories of the p-factor [52].

The finding of the moderating effect of the family risk profile on the relationship between age of onset of disruptive behaviour and rule-breaking problems during adolescence can enhance insight into this unique but small group of adolescents who have experienced risk factors primarily in the family domain. In a previous study, adolescents with this risk profile with mainly family risks were found to have an elevated risk of offending behaviour after discharge from secure residential care [53]. This was opposite to expectations of a lower risk of offending because of their low number of risk factors, less comorbid psychopathology, and a lower occurrence of criminal history. The findings of the present study that the childhood-onset group differs from the adolescence-onset group according to their rule-breaking behaviour at the start of admission evokes the explanation that the group of adolescents with the risk profile with mainly family risks may be still somewhat heterogeneous. For now, the findings confirm that this is a unique group that needs extra attention during residential treatment. As the impact of family risk factors in these adolescents, such as childhood history of maltreatment and criminal behaviour of family members, turns out to be large and persistent; further, previous interventions have proved insufficient [1]. Therefore, the importance of early and intensive attachment focused, family-based interventions also appears from this study, both for children living with their biological parents and for children living with foster parents. For children who were already placed in foster care, it is extremely important that they can grow up in a safe and stable foster family. The children with early-onset disruptive behaviour who experienced mainly family risk factors must be detected in the primary health care facilities. Especially in this group, the therapeutic intervention must take place earlier, for example through attachment focused, family-based interventions or parent management training [54, 55].

A major strength of this study is the focus on heterogeneity in the oft-examined relationship between age of onset and externalising behaviour. To move beyond the possible regression to the mean in this relationship, the influence of distinct subgroups—which are four risk profiles from a previous person-centered study [29]—on the relationship between age of onset and rule-breaking and aggressive behaviour was explored. Nevertheless, this study is not without limitations. First, a cross-sectional design was used; the possibility therefore exist that other factors (e.g., social and economic pressures or the psychiatric vulnerability), as predicted by family stress models [56] were the cause of both the presence of risk factors and the onset of problem behaviour. Second, because this study only considered male patients at one hospital for youth forensic psychiatry and orthopsychiatry, generalisability is limited. However, since this hospital offers treatment to a specific group of adolescents with major psychiatric and behavioural problems from all over the country, the sample of our study probably is representative of the population of adolescents with severe problems in the Netherlands. Third, we only used reports from professional caregivers. This was a deliberate choice and flowed from the fact that the risk profiles and age of onset information also were based on proxy reports. Because the average correlation between proxy reports and self-reports is very low [57], the assumption was that the moderator effect for the self-reports were only small. Fourth, the item ‘onset of problem behaviour’ of the JFP was used to operationalise age of onset, in which the transition to high school (i.e., the age of 12) was used as cut-off between childhood and adolescence onset. This deviated from the 10-years cut-off point for childhood onset as described in the DSM-5 and ICD-11. In future research, information could possibly be collected differently—prospectively instead of retrospectively—so that information can be based on a prospective measure for age of onset (with another cut-off point) and on reports from multiple informants including the adolescents themselves. In addition, future research could focus on larger samples in multiple institutions so that the risk profiles and the found moderator effect can be replicated.

## Conclusion

The heterogeneity of externalising behaviour among adolescents admitted to secure residential care is established by finding a moderating effect of risk profiles on the relation between age of onset of disruptive behaviour and rule-breaking behaviour. While respecting the limitations of this study, it can be preliminarily concluded that adolescents with a profile with mainly family risk factors and childhood-onset disruptive behaviour appear to be distinctive in terms of rule-breaking behaviour from those with the same profile but with adolescence-onset disruptive behaviour. According to the cumulative risk hypothesis, the presence of many risk factors in more domains had no added value in explaining externalising problems beyond the age of onset. The causal pathways of externalising problems, however, are varied and complex. Further research is necessary; in particular, longitudinal prospective research in larger samples is recommended.

## Data Availability

The datasets analysed during the current study are not publicly available due to intellectual property rights, but are available from the corresponding author on reasonable request.

## Abbreviations

CBCL: Child behavior checklist
DSM-IV-TR: Diagnostic and statistical manual of mental disorders, fourth edition, text revision
IRB: Institutional Review Board
JFP: Juvenile forensic profile
LCA: Latent class analysis
ROM: Routine outcome monitoring
SAVRY: Structured assessment of violence risk in youth

## Footnotes

1 For reasons of brevity, the term ‘adolescent’ is used throughout the text; this also includes young adults (18–23 years) who were sentenced under the Dutch juvenile justice system.
